# Assessment of knowledge, perceptions, and readiness of healthcare professionals towards clinical pharmacogenomics implementation in Qatar: a mixed-method study

**DOI:** 10.1080/20523211.2024.2429785

**Published:** 2024-11-26

**Authors:** Shaikha Jabor Alnaimi, Fatima Ajaj, Ahmed Awaisu, Turfa Alhathal, Shaban Mohammed, Moza Alhail

**Affiliations:** a Pharmacy Department, Hamad Medical Corporation, Doha, Qatar; b Department of Clinical Pharmacy and Practice, College of Pharmacy, Qatar University, Doha, Qatar

**Keywords:** Pharmacogenomics, mixed method, survey, focus groups, perceptions

## Abstract

**Introduction:**

Pharmacogenomics implementation in clinical practice is anticipated to improve our understanding of individual variations in drug response and optimise the safety and efficacy of drug therapy. We aimed to assess the knowledge, perceptions, and readiness of physicians, pharmacists, and nurses in Qatar regarding the implementation of clinical pharmacogenomics.

**Methods:**

A mixed-method study with an explanatory sequential design was conducted. Phase I was the quantitative phase which involved sending an online survey to physicians, pharmacists, and nurses. Phase II was the qualitative phase which involved conducting focus group discussions.

**Results:**

A total of 802 responses were collected, with a response rate of 20%. Only 15.4% of participants had previous pharmacogenomics-related training. The median knowledge score for healthcare professionals was 4 out of 10 denoting low level of knowledge. However, compared to other professions, pharmacists had a higher knowledge score (*p*-value <0.001) and Doctor of Pharmacy (PharmD) holders scored higher than BSc holders (*p*-value <0.001). Despite the low level of knowledge, perceptions of healthcare professionals were positive. In addition, the majority believed knowledge of pharmacogenomics is necessary and that counselling patients on pharmacogenomics requires specialised training pharmacogenomic principles in practice. The main themes extracted from the focus group discussions were knowledge, outcome expectations, preparedness, facilitators, barriers, public education, and implementation planning. Regarding readiness, most healthcare professionals reported that they are not currently confident in applying

**Conclusions:**

Healthcare providers have a low level of knowledge of pharmacogenomics. Despite this, the majority have positive perceptions towards its implementation in practice. Compared to other professionals, pharmacists with a PharmD degree scored higher in the knowledge assessment. Most healthcare providers report low confidence regarding the readiness for the implementation of pharmacogenomics and report a lack of knowledge, specialised training, and clinical guidelines as barriers.

## 1. Introduction

Pharmacogenomics is the study of the effect of human genetic variability on the safety and efficacy of drug therapy (Maitland-van der Zee et al., 2000). The advent of whole genome sequencing is anticipated to revolutionise the application and clinical utility of pharmacogenomics by providing valuable insights into drug metabolism and response (Nickola et al., 2012). As the move towards precision medicine grows globally, several healthcare and research institutions fund pharmacogenomics research with the intention to understand the genetic links to interindividual variability in response to drug therapy. In the United Kingdom (UK), the 100,000-genome project supports research studies with the overall goal of implementing pharmacogenomic data in clinical practice (Samuel & Farsides, 2017). In the United States (US), steps have been taken to establish pharmacist-led pharmacogenomics clinics in primary care (Krebs & Milani, 2019; Weinstein et al., 2019).

As pharmacogenomics aims at individualising drug therapy according to the presence of genetic biomarkers, its implementation is expected to improve efficacy, reduce adverse effects, and improve the cost-effectiveness of treatments (Sadee et al., 2023). However, there are documented barriers to the implementation of pharmacogenomics in clinical practice. These include competence and readiness of healthcare professionals, availability of resources, and cultural and religious issues (Sadee et al., 2023). In a cross-sectional survey to assess the knowledge of physicians and pharmacists about pharmacogenomics, the overall mean knowledge score was low with no significant difference between physicians and pharmacists (Albassam et al., 2018). Furthermore, only 16% of participants expressed confidence in applying pharmacogenomics in their practice settings. The main barriers to pharmacogenomics implementation were the lack of education or training and the lack of clinical guidelines (Albassam et al., 2018). Rafi et al. conducted a qualitative study to investigate the potential barriers, opportunities, and challenges associated with integrating pharmacogenomics into primary care in the UK. The key concerns identified through a thematic analysis were the utility of pharmacogenomics in primary care, strategies for educating the primary care workforce, ethical, legal, and social considerations, and the potential economic impact on the healthcare system (Rafi et al., 2020).

To ensure the efficient utilisation of pharmacogenomic testing and application in clinical practice, it is crucial to understand healthcare professionals’ acceptance, competence, and readiness for its implementation. Nationally, there is a lack of published literature assessing the competence and readiness of healthcare professionals in Qatar. Therefore, this study aimed to evaluate Qatar healthcare professionals’ knowledge, perceptions, and readiness regarding pharmacogenomics implementation in clinical practice. Additionally, the study sought to identify barriers to its application in their practice settings and the preferred learning format for future professional development and training.

## 2. Method

### 2.1. Study setting and population

The study was conducted at Hamad Medical Corporation (HMC), the main public healthcare institution that provides secondary and tertiary care to the residents of Qatar. There are twelve hospitals under HMC that provide either general or specialised care. Healthcare professionals come from various countries and the majority have completed their education abroad. Ethics approval was obtained from the Medical Research Center, Hamad Medical Corporation (MRC-01-21-126).

### 2.2. Study design and data collection

We conducted a mixed-method study with an explanatory sequential design between March 2022 and September 2023. The two-phase approach involved initially collecting and analysing quantitative data, followed by collecting and analysing qualitative data, where the latter served the purpose of providing deeper insights into the quantitative results. In Phase I, we used convenience sampling by sending the survey to a reachable population. The reachable population were healthcare professionals who were part of a mail group, this allowed us to identify the total number of recipients as 4019 healthcare professionals. Given an overall reachable population of 4019 healthcare professionals, a 99% confidence interval (CI), and a 5% margin of error, the required sample size is 570 participants. Emails were initially sent every 2 weeks for the first month and then weekly reminders were sent for another month. In Phase II, we used purposive stepwise sampling to ensure representativeness and to minimise selection bias. We invited participants from across different facilities, professions, and specialties. Initially, we asked individuals to indicate their interest in participating in the discussions. Among those, we then selected individuals with sufficient years of experience (above 5 years), preferably providing care to patients with cardiovascular disease and cancer in which personalised medicine is more established, and those who may have relevant experience in it. However, if the number of invitees per focus group session was less than 6, other professionals with less than 5 years of experience and from different specialties were invited.

### 2.3. Quantitative phase

A questionnaire was initially developed using questions adapted from previously published tools (Albassam et al., 2018; Elewa et al., 2015; Rahma et al., 2020). Subsequently, the survey was reviewed by five experts, 3 pharmacy academics, and 2 physicians with research experience in pharmacogenomics, for content validity. It was then reviewed by 10 healthcare professionals for face validity. This review aimed to enhance clarity and gather feedback. Furthermore, a pilot phase involving 30 healthcare professionals was conducted to identify and address any potential gaps, including clarity, lack of ambiguity, and time burden. The survey was divided into seven sections, including demographics (12 items), training/education (7 items), knowledge (10 items), perceptions (12 items), readiness (6 items), future education (4 items), and barriers (1 item).

The knowledge section of the survey included ten fact-based questions with the three following choices (True, false, do not know/not sure). Each correct answer was assigned 1 point, while each incorrect or ‘do not know/not Sure’ was assigned 0 points. A total knowledge score of 10/10 indicates that all questions were answered correctly. A score that is > 6/10 was considered high and a score ≤6/10 was considered low, based on a similar previous study conducted in Kuwait (Albassam et al., 2018). The fourth section of the survey consisted of twelve questions assessing perceptions towards the implementation of pharmacogenomics in clinical practice. Participants were asked to rate the extent to which they agreed with each statement, using a 5-point Likert scale (i.e. strongly agree, agree, neutral, disagree, and strongly disagree). The fifth section of the survey consisted of six questions assessing readiness for applying pharmacogenomics in clinical practice. Participants were asked to give a score from 0–4 to each statement to describe their level of confidence where 0 = not at all confident and 4 = extremely confident. The last section of the survey asked participants to indicate barriers to the implementation of pharmacogenomics in clinical practice using a predefined list, where participants could select more than one option, and a free-response section was available to capture any other items.

Internal consistency reliability for items under knowledge, perceptions, and readiness to clinical pharmacogenomics implementation sections was tested by Alpha (Cronbach’s) and was equal to 0.912, 0.721, and 0.917 respectively. The final version of the questionnaire was administered through the SurveyMonkey platform. Each participant was restricted to taking the survey only once, and participation was voluntary.

### 2.4. Qualitative phase

The qualitative part of the study was conducted using focus group discussions. Participants who expressed interest in the survey to participate in the focus groups were sent an email invitation including the details of the study, the time and duration of the focus group discussions, and an informed consent form. To avoid logistic challenges among the participants, the focus group discussions were conducted virtually through Microsoft Teams. Each session lasted between 60 and 90 min. Focus group sessions were arranged to include either participants from the same profession or multidisciplinary participants in the same session. The sessions were conducted in English and moderated by two research team members, using an interview guide that included eight open-ended questions and prompts. The focus group guide was prepared by the research team members based on study objectives and previous literature and was revised by a member with expertise in the field of qualitative research. Discussions were audio recorded and subsequently transcribed verbatim by two of the research team members.

### 2.5. Data analysis

Data from the quantitative phase were checked for completeness, coded, and analysed. Continuous variables were summarised as median and interquartile range (IQR) while categorical variables were reported as frequencies and proportions. For the assessment of normality, the Kolmogorov–Smirnov (K-S) test was used. The total knowledge score was found to be non-normally distributed (*p* value = 0.000) and non-parametric tests were used for inferential statistics. Independent samples Man-Whitney U test was used to compare knowledge scores. The Kruskal–Wallis test was used to examine the relationship and to identify significant differences in knowledge scores between the independent groups. Statistical significance was considered at a *p*-value <0.05. Data analysis was done using the Statistical Package for Social Sciences (SPSS) IBM Version 29. EndNote 21 was used to manage references.

For the focus group discussions, manual thematic analysis was applied to generate the themes to ensure a transparent and in-depth analysis of the data. Also, because the data generated from the qualitative phase was not large, Microsoft Word was to label the codes and organise the generated themes. Coding was undertaken by two researchers and any discrepancy was resolved by discussion with other team members. During the analysis, the initial themes were derived based on the researchers’ insight as well as a previous study that explored the same scope (Rahma et al., 2020). Additional themes were derived during the analysis and classified as emergent themes if the ideas discussed by the participants were not covered by the pre-defined themes.

## 3. Results

### 3.1. Quantitative phase

#### 3.1.1. Demographic characteristics

The survey was sent to healthcare professionals who were members of mail groups. The total number of members was 4019 of these 802 responded to the survey (response rate 20%). Out of which, 63.2% were females. Around half of the participants were nurses (54.1%), followed by pharmacists (26.1%), and physicians (19.8%). Most of the participants had at least 10 years of practice experience (75.9%). The demographic and professional characteristics of participants are presented in (Table 1).

**Table 1.** The demographic characteristics of healthcare participants (*N* = 802).

Characteristics	*n* (%)
**Gender**	
Female	501 (63.2)
Male	292 (36.8)
**Profession**	
Pharmacist	209 (26.1)
Physician	159 (19.8)
Nurse	434 (54.1)
**Level of practice**	
Pharmacist	
Staff pharmacist	119 (59.2)
Clinical pharmacist	39 (19.4)
Clinical pharmacy specialist	6 (3.0)
Assistant director of pharmacy	6 (3.0)
Director of pharmacy	1 (0.5)
Other	30 (14.9)
Physician	
Resident	15 (9.6)
Specialist	43 (27.4)
Fellow	7 (4.5)
Consultant	80 (51.0)
Other	12 (7.6)
Nurse	
Staff nurse	317 (73.7)
In-charge nurse	45 (10.5)
Clinical nurse	13 (3.0)
Head nurse	29 (6.7)
Other	26 (6.0)
**Area of specialty**	
Not applicable	71 (10.3)
Cardiology	35 (5.1)
Infectious disease	12 (1.7)
Internal medicine	63 (9.1)
Obstetrics/gynecology	132 (19.1)
Oncology/hematology	29 (4.2)
Pediatrics	62 (9.0)
Psychiatry	24 (3.5)
Other	264 (38.2)
**Highest academic qualification**	
Bachelor’s	420 (57.0)
PharmD	35 (4.7)
MD	72 (9.8)
MSc	95 (12.9)
PhD	19 (2.6)
Other	96 (13.0)
**Additional professional qualifications**	
None	381 (47.5)
Residency	42 (5.2)
Fellowship	49 (6.1)
Board-certification	135 (16.8)
Postgraduate certificate or diploma	166 (20.7)
**Years of practice since graduation**	
Less than 5 years	69 (9.4)
5–9 years	108 (14.8)
10–14 years	215 (29.4)
15–19 years	125 (17.1)
20 years and above	215 (29.4)

Note: PharmD: Doctor of Pharmacy, MD: Doctor of Medicine, MSc: Master of Science, PhD: Doctor of Philosophy.

#### 3.1.2. Pharmacogenomics training and application

Participants were asked questions to assess prior pharmacogenomic training/education and its application in clinical practice. The majority of the participants (84.6%) reported that they had not completed any pharmacogenomics-related training or education.

#### 3.1.3. General knowledge of pharmacogenomics

The median (IQR) knowledge score was [4 (6) out of 10], indicating a low level of knowledge of pharmacogenomics. There was no significant difference in the total knowledge score between female and male participants [4 (6) vs. 4 (6)], *p*-value 0.833. However, pharmacists had a significantly higher knowledge score compared to physicians and nurses [5 (6) vs. 4 (6) vs. 3 (5)], respectively, *p*-value <0.001. Among pharmacists, participants with a Doctor of Pharmacy (PharmD) degree, had a significantly higher total knowledge score [6 (4)] compared to participants with other qualifications (*p*-value <0.001). Additional comparisons in the median knowledge score across different demographics are presented in (Table 2). Overall, the assessment of knowledge shows that 84.5% of healthcare providers recognise the role of pharmacogenomics in individualising drug therapy, and 82.5% agree it can improve medication efficacy. However, only 41.3% understand that testing is not available for most medications. Knowledge about the role of specific genes is lower, for example, only 36.5% recognise the role of CYP2C9 polymorphism on the metabolism of clopidogrel. A summary of the results of the general knowledge questions is presented in (Table 3).

**Table 2.** The median knowledge score among health care professionals (*N* = 802).

	Variable (*N*)	Median knowledge score (IQR)	*P*-value
**Gender**	Male (290)	4.0 (6)	0.833
	Female (500)	4.0 (6)	
**Profession**	Pharmacist (207)	5.0 (6)	.000
	Physician (159)	4.0 (6)	
	Nurse (433)	3.0 (5)	
**Area of specialty**	Not applicable (70)	5.0 (6)	0.054
	Cardiology (35)	5.0 (4)	
	Infectious disease (12)	5.5 (5.75)	
	Internal medicine (62)	4.0 (3.25)	
	Obstetrics/Gynecology (110)	4.0 (6)	
	Oncology/Hematology (29)	4.0 (7)	
	Pediatrics (62)	4.0 (6)	
	Psychiatry (24)	0 (5.75)	
	Women's Health (22)	1.5 (5.25)	
	Other (263)	4.0 (6)	
**Highest academic qualification**	Bachelor’s (418)	4 (6)	<.001
	PharmD (34)	6 (4)	
	MD (72)	4 (6)	
	MSc (95)	4 (6)	
	PhD (19)	5 (7)	
	Other (96)	4 (5)

**Table 3.** General knowledge of pharmacogenomics and its application in clinical practice (*n* = 802).

	Correct answer	True *n* (%)	False *n* (%)	Not sure *n* (%)
An individual’s genetic characteristics for a drug response constantly change over their lifetime.	False	283 (52.0)	127 (23.3)	134 (24.6)
Pharmacogenomic testing is currently available for the majority of medications used in clinical practice.	False	225 (41.3)	129 (23.7)	191 (35.0)
Pharmacogenomics has an important role in individualising patients’ drug therapy.	True	458 (84.5)	17 (3.1)	67 (12.4)
Pharmacogenomics does not have a role in identifying drug-drug interactions.	True	402 (74.3)	49 (9.1)	90 (16.6)
Pharmacogenomic information is currently included in the package insert of some medications	True	280 (51.8)	55 (0.2)	206 (38.1)
The application of clinical pharmacogenomics can improve the efficacy of several medications.	True	448 (82.5)	17 (3.1)	78 (14.4)
The application of clinical pharmacogenomics is not cost-effective, because pharmacogenomic testing is expensive.	False	144 (26.6)	161 (29.7)	237 (43.7)
Currently, there are pharmacogenomic resources that can guide clinical decision-making.	True	346 (64.0)	29 (5.4)	166 (30.7)
The most important gene affecting warfarin dosing among different populations is the CYPY4F2.	False	176 (32.4)	85 (15.7)	282 (51.9)
Pharmacogenomic studies have shown that CYP2C9 polymorphism has the most pronounced effect on clopidogrel metabolism.	True	197 (36.5)	40 (7.4)	303 (56.1)

#### 3.1.4. Perceptions towards the implementation of pharmacogenomics in clinical practice

Participants’ perception towards the implementation of pharmacogenomics in clinical practice is presented in (Table 4). More than half of the participants tend to agree

**Table 4.** Perceptions towards the implementation of pharmacogenomics in clinical practice (*N* = 802).

	Disagree *n* (%)	Neutral *n* (%)	Agree *n* (%)
I am aware of the challenges associated with integrating pharmacogenomics into routine clinical practice.	29 (5.8)	131 (26.3)	339 (67.9)
All healthcare professionals should have good knowledge of pharmacogenomics.	22 (4.4)	64 (12.8)	413 (82.8)
Pharmacogenomic testing conflicts with my cultural and/or religious beliefs.	203 (40.7)	146 (29.3)	150 (30)
The evidence of the clinical use of pharmacogenomics supports routine pharmacogenomic testing.	26 (5.2)	149 (29.9)	324 (64.9)
Pharmacogenomic testing should be applied in my clinical practice.	12 (2.1)	117 (23.4)	370 (74.1)
Implementation of pharmacogenomics in clinical practice requires multidisciplinary efforts.	9 (1.8)	67 (13.4)	423 (84.8)
It is my role to be able to provide information on appropriate use of pharmacogenomic testing to other healthcare professionals and/or patients.	41 (8.2)	122 (24.4)	336 (67.3)
Counselling patients about pharmacogenomics requires specialised training.	6 (1.2)	64 (12.8)	429 (86)
Regulatory drug agencies should create a list of approved drugs containing pharmacogenomics-related information and recommendations.	10 (2)	77 (15.4)	412 (82.6)
Healthcare professional education programmes (e.g. schools of medicine, schools of pharmacy, schools of nursing … etc.) should make it mandatory to include pharmacogenomics content in their curricula.	19 (3.8)	87 (17.4)	393 (78.8)
International healthcare accreditation agencies (e.g. JCI) should create guidelines to guide pharmacogenomics implementation in practice.	13 (2.6)	103 (20.6)	383 (67.8)
I would have concerns about data security and protection of personal information associated with pharmacogenomic testing.	41 (8.2)	127 (25.5)	331 (66.3)

Note: JCI: Joint Commission International.

with all aspects of the implementation of pharmacogenomics in clinical practice including awareness of the challenges and responsibilities of health care professionals. The majority (74.1%) of the participants agreed that pharmacogenomic testing should be applied in their clinical practice. Factors highly perceived to contribute to the implementation of pharmacogenomics in clinical practice were knowledge, multidisciplinary team efforts, specialised training, and the incorporation of pharmacogenomics recommendations by regulatory drug agencies. However, perceptions varied regarding pharmacogenomic testing and whether it conflicts with cultural and/or religious beliefs.

#### 3.1.5. Readiness for pharmacogenomics

**Figure 1.**
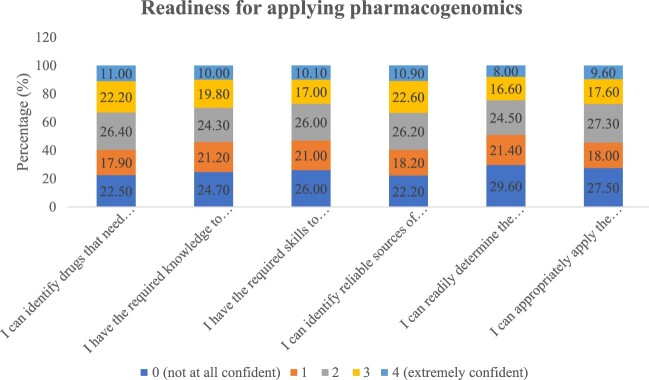
Confidence levels for applying pharmacogenomics in practice.

The readiness for applying pharmacogenomics in practice settings was assessed by measuring the confidence level (where 0 = not at all confident and 4 = extremely confident) (Figure 1). The majority of participants tend to report low confidence or neutral in all items, including knowledge, skills, identification of reliable resources, and application of testing results. About 10% of the participants reported extreme confidence in all items.

#### 3.1.6. Future education

Insights on future education about pharmacogenomics were also surveyed. The highest selected modality of education was continuous professional development programmes (39.2%) followed by an academic certificate or diploma (25.3%) (Table 5). Moreover, 83.3% of the participants expressed interest in taking a pharmacogenomics course or training in a face-to-face setting (25.2%) or online-based learning (30.4%). The most reported priority areas to implement pharmacogenomics were cardiovascular disease (34.5%), cancer (33.0%), and pain management (32.8%).

**Table 5.** Future education about pharmacogenomics (*N* = 802).

	*n* (%)
Q31. How do you wish/prefer the pharmacogenomics training to be incorporated (choose all that apply)?	
Undergraduate curriculum	111 (13.8)
Residency programmes	121 (15.1)
Fellowship programmes	91 (11.3)
Continuous professional development programmes	314 (39.2)
Certificate or diploma	203 (25.3)
Q32. Are you interested in taking a pharmacogenomics course or training?	
Yes	385 (83.3)
No	28 (6.1)
Not sure	49 (10.6)
Q33. If your answer is ‘yes’ to the above question, which method of education delivery do you prefer to learn about pharmacogenomics? Please tick (√) all that applies.	
Face-to-face approach	202 (25.2)
Online-based learning	244 (30.4)
Self-directed learning	83 (10.3)
Blended learning (i.e. face-to-face and online activities)	148 (18.5)
Q34. Which areas do you believe are a priority to implement pharmacogenomics in Qatar? Tick (√) all that applies.	
Mental health	211 (26.3)
Cardiovascular diseases	277 (34.5)
Cancer	265 (33.0)
Pain management	263 (32.8)
Endocrine diseases	242 (30.2)

#### 3.1.7. Barriers to the application of pharmacogenomics

**Figure 2.**
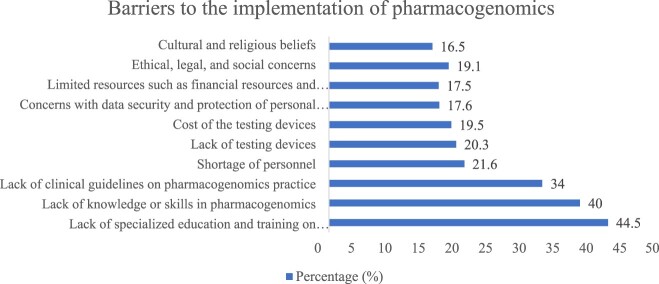
Barriers to the application of pharmacogenomics.

When asked about perceived barriers to the application of pharmacogenomics, the top three were lack of specialised education and training (44.5%), lack of knowledge or skills (40%), and lack of clinical practice guidelines (34%) (Figure 2).

### 3.2. Qualitative phase

Six focus group discussions were conducted, two of them were arranged to include participants with the same profession, one group for pharmacists, and the other group included only nurses. The remaining four focus group discussions were multidisciplinary. Data saturation, where no new information or ideas were provided by the participants, was deemed to be achieved at the sixth focus group session. Details about the total number of participants and their demographics are presented in (Table 6). The majority of the participants were females above 30 years old with a Bachelor’s (BSc) degree as the highest academic qualification. Out of the total 31 participants, 15 (48%) were pharmacists, 14 (45%) were nurses and only 2 (6.4%) were physicians.

**Table 6.** Demographic characteristics of the focus group participants (*N* = 31).

Characteristics	*n* (%)
**Gender**	
Female	21 (67.7%)
Male	10 (32.3%)
**Age group**	
20–30	5 (16%)
More than 30	26 (84%)
**Profession**	
Pharmacists	15 (48.3%)
Physicians	2 (6.4%)
Nurses	14 (45.1%)
**Level of practice**	
Consultant	2 (6.45%)
Clinical pharmacist	4 (12.9%)
Staff pharmacist	11 (35.4%)
Staff nurse	12 (38.7%)
other	2 (6.4%)
**Area of specialty**	
Oncology/Hematology	5 (17.2%)
Pediatrics	4 (13.7%)
Internal medicine	3 (10.3%)
Other	19 (61.2%)
**Highest academic qualification**	
Bachelor’s	19 (61.2%)
MSc	6 (19.3%)
PharmD	3 (9.6%)
Other	3 (9.6%)
**Years of practice since graduation**	
Less than 5 years	5 (16.1%)
5–9 years	7 (22.5%)
10–14 years	7 (22.5%)
15–19 years	8 (25.8%)
20 years and above	4 (12.9%)

Several themes and sub-themes emerged from the analysis which are presented in (Table 7). Some of the themes were predominantly discussed by participants during multiple sessions. These included: knowledge about pharmacogenomics, expectations about outcomes, and facilitators and barriers to implementation. The emerging theme was the implementation plan.

**Table 7.** Major themes and subthemes with illustrative quotes.

Theme	Sub-theme	Illustrative quote(s)
**Knowledge**	- Knowledge about pharmacogenomics - Source of knowledge - Training and education	‘This is a very new topic (pharmacogenomics) … In general, I know it's something related to tailoring the medication for certain patients according to their genetics.’ (FG1, Pharmacist) ‘I have a degree in genomics, part of it is pharmacogenomics but we don’t really practice it … ’ (FG4, Physician) ‘I have 2 publications in the pharmacogenomics field; one is a case report and the other one about warfarin drug-gene interactions’ (FG3, Pharmacist) ‘We need training with some practical application and mandatory education.’ (FG2, Nurse)
**Expectations about outcomes**		‘It will minimize the side effects of drugs, and we will be able to know what dose is suitable for each individual.’ (FG6, Nurse) ‘Definitely, if you apply pharmacogenomics there will be improved outcomes and efficacy of medications, because it is tailored to that specific patient with all his genetic background.’ (FG5, Physician)
**Preparedness**		‘I think we are more than ready, we just need to know how to apply it then we need to prepare for its implementation’ (FG1, Pharmacist) ‘I don't think that HMC is ready in my opinion, because the staff are not trained or educated about genomics and the testing process’ (FG2, Nurse)
**Facilitators to implementation**		‘There are some universities in Qatar or internationally that offer pharmacogenomics courses’ (FG1, Pharmacist) ‘I think HMC is ready to start, especially with the collaboration with Qatar Genome, which will facilitate testing’ (FG6, Pharmacist)
**Barriers to implementation**	- Cultural influences - Knowledge of healthcare professionals - Cost	‘We also find a lot of challenges with patients especially when we are introducing something new, they usually raise a lot of queries, and each of them will have their own cultural beliefs’ (FG2, Nurse) ‘ … if it was explained properly and the key message was delivered to the patient in a proper way, there will be no barrier for anything unless we miscommunicate’ (FG5, physician) ‘I think the biggest barrier is the lack of knowledge’ (FG1, Pharmacist) ‘The cost of establishing a new service could be a barrier in initiating the service’ (FG6, Pharmacist)
**Public education**		‘One of the most important things that we need to work on … is raising the awareness regarding the pharmacogenomics among patients or the society because this concept is still not well known and I think patients should be exposed to this idea at early stage … before coming and attending such clinics or such service.’ (FG6, Pharmacist).
**Implementation plan**	- Input from experts in the field - Implementation approach	‘Maybe bringing some experts from institutions that already have pharmacogenomic implemented and have some experts from time to time or during the initial process to supervise and guide the staff … ’ (FG3, pharmacist) ‘I'm not sure how this service will be provided, will it be a consultation service in which a group of patients attend to know how to manage their medications?’ (FG3, pharmacist)

#### 3.2.1. Knowledge

This theme addresses the level of knowledge among healthcare professionals about the practice of pharmacogenomics and what factors contribute to this knowledge. Under this theme, the sub-themes that emerged are the level of knowledge, source of knowledge, and training courses.

##### 3.2.1.1. Knowledge about pharmacogenomics

Many participants expressed that they were familiar with the concept but do not know how it is applied in practice. One pharmacist mentioned:

This is a very new topic (pharmacogenomics) … In general, I know it's something related to tailoring the medication for certain patients according to their genetics. (FG1, Pharmacist)

##### 3.2.1.2. Source of knowledge

On the other hand, few participants showed interest in this concept and reported that they obtained training courses to learn more about pharmacogenomics:

I have a degree in genomics, part of it is pharmacogenomics but we don’t really practice it … . (FG4, Physician)

Another participant mentioned that his special interest led him to focus on pharmacogenomics research:

I have 2 publications in the pharmacogenomics field; one is a case report and the other one about warfarin drug-gene interactions. (FG3, Pharmacist)

##### 3.2.1.3. Training and education

After a general agreement among the staff about the limited knowledge about the application of pharmacogenomics, many of them elaborated that training and education of healthcare professionals is an essential initial step. For example, one physician mentioned:

Education is the most important part. But I think if you can do it in a specialty context, so that each specialty has a specific training program directed to them. This will be easier to understand. Of course, there will be a general session, but specialty-specific training might be beneficial. (FG4, Physician)

Another nurse emphasised the importance of providing educational courses:

We need training with some practical application and mandatory education. (FG2, Nurse)

#### 3.2.2. Expectations about outcomes

There was a general consensus about the importance of implementing pharmacogenomics in practice and many participants mentioned that it will be of great benefit to patient care in terms of improving the safety and efficacy of medications:

It will minimize the side effects of drugs, and we will be able to know what dose is suitable for each individual. (FG6, Nurse)

Definitely, (with) pharmacogenomics there will be improved outcomes and efficacy of medications, because it is tailored to that specific patient with all his genetic background. (FG5, Physician)

#### 3.2.3. Preparedness

We had mixed results regarding the level of preparedness to establish pharmacogenomics in practice. Some participants mentioned that their institution is ready in terms of infrastructure and personnel, and they only require guidance on its application. On the other hand, others believe that the lack of knowledge and training is substantial and is the main reason why the institution is not yet prepared for implementation. Some examples include:

I think we are more than ready, we just need to know how to apply it … and prepare for its implementation. (FG1, Pharmacist)

I don't think that HMC is ready in my opinion, because the staff are not trained or educated about genomics and the testing process. (FG2, Nurse)

#### 3.2.4. Facilitators to implementation

In a discussion about what might facilitate the implementation, several ideas have been discussed such as access to academic courses, support of the higher administration, and availability of institutions specialised in genomics in Qatar. Examples include:

There are some universities in Qatar or internationally that offer pharmacogenomics courses. (FG1, Pharmacist)

I think HMC is ready to start, especially with the collaboration with Qatar Genome, which will facilitate testing. (FG6, Pharmacist)

#### 3.2.5. Barriers to implementation

Many factors were considered barriers to establishing clinical pharmacogenomics in Qatar which required classification into sub-themes:

##### 3.2.5.1. Cultural influence

The role of the patient’s culture in the acceptance of genetic testing and the use of genetic information in healthcare was controversial. Some participants believe that healthcare professionals may face challenges in accepting clinical pharmacogenomics by society, especially during the early stages of implementation. One of the nurses discussed this point:

We also find a lot of challenges with patients especially when we are introducing something new, they usually raise a lot of queries, and each of them will have their own cultural beliefs. (FG2, Nurse)

On the other hand, some stated that with proper awareness and communication it would be easier for patients to accept any new practice:

… if it was explained properly and the key message was delivered to the patient in a proper way, there will be no barrier for anything unless we miscommunicate. (FG5, physician)

##### 3.2.5.2. Knowledge of healthcare professionals

The majority of participants agreed that lack of knowledge about pharmacogenomics among healthcare professionals is one of the main barriers to implementation:

I think the biggest barrier is the lack of knowledge. (FG1, Pharmacist)

##### 3.2.5.3. Cost

Some concerns were raised about the financial impact of using pharmacogenomics as a result of using specialised testing:

The cost of establishing a new service could be a barrier in initiating the service. (FG6, Pharmacist)

#### 3.2.6. Public education

Many participants discussed the importance of raising awareness among society about pharmacogenomics among society to facilitate acceptance among patients:

One of the most important things that we need to work on … is raising the awareness regarding the pharmacogenomics among patients or the society because this concept is still not well known and I think patients should be exposed to this idea at an early stage … before coming and attending such clinics or such service. (FG6, Pharmacist)

#### 3.2.7. Implementation plan

Many participants were motivated by the idea of establishing pharmacogenomics services in Qatar and they mentioned some strategies that might be considered during the implementation process. We classified these ideas into two sub-themes: input from experts in the field and the implementation approach.

##### 3.2.7.1. Input from experts in the field

Some participants preferred to have input from institutions that have already established the service of pharmacogenomics in their practice, and they highlighted its importance during the training and when initiating the service. A pharmacist mentioned:

… bringing some experts from institutions that already have pharmacogenomic implemented and have some experts from time to time or during the initial process to supervise and guide the staff … . (FG3, pharmacist)

##### 3.2.7.2. Implementation approach

Many participants raised some concerns about the appropriate approach to integrate pharmacogenomics in daily patient care. Some of them provided suggestions to be considered during the process of implementation. For example, offering point-of-care testing at clinics, integrating pharmacogenomic data in electronic medical records, and establishing a consultation service:

I'm not sure how this service will be provided, will it be a consultation service in which a group of patients attend to know how to manage their medications? (FG3, pharmacist)

## 4. Discussion

The advent of whole genome sequencing can revolutionise precision medicine by providing valuable insights into the interindividual variability of response to drug therapy (Sadee et al., 2023). In our mixed-method study, we aimed to benchmark the current level of knowledge, barriers, opportunities, and challenges associated with integrating pharmacogenomics into the tertiary care setting in Qatar. Despite the current low level of knowledge about pharmacogenomics reported in this study, participants acknowledged the growing importance of pharmacogenomic testing and recognised the need to address the practical implications of incorporating genomic information in patient care. This study also highlights issues related to the education of healthcare professionals and patients, which must be considered when planning the integration into clinical practice.

A study in Kuwait that included pharmacists and physicians reported that only a minority of respondents (8.9%) had received education or training in pharmacogenomics (Albassam et al., 2018). In that study, the mean knowledge score was 45% without a significant difference between pharmacists and physicians. The findings of our study showed a gap in the practical knowledge of healthcare professionals related to pharmacogenomics. Although PharmD holders scored higher in the survey, they also agreed during focus group discussions on the need for training and education before the clinical implementation. These findings are consistent with previous studies conducted globally, indicating a knowledge gap and a need for pharmacogenomics training and education among healthcare professionals (Abdela et al., 2017; Imam et al., 2022; Taber & Dickinson, 2014; Veilleux et al., 2020).

Despite the low level of knowledge, respondents in our study expressed positive perceptions towards pharmacogenomics and its application, as more than half agreed on questions related to positive perceptions. This was also reflected in focus group discussions when expectations about outcomes were discussed, as the majority of the participants agreed on the importance of using pharmacogenomics in improving patient outcomes and minimising harm. Understandably, however, only 10% reported extreme confidence in the application of pharmacogenomics, denoting low levels of readiness. While the focus group discussions reveal some contrasting perceptions of readiness, participants who believed that their institution was ready, still reported knowledge as the main obstacle. Meanwhile, many focus group participants think that their institution is not yet prepared for the implementation and highlighted that strong infrastructure, support from stakeholders, and education are key prerequisites.

Of interest, a similar study that was conducted to assess the knowledge and confidence of pediatricians in the US and Japan reported that despite that >75% of physicians received prior education in genetics, <10% reported familiarity with the term pharmacogenomics (Rahawi et al., 2020). Despite their respondents being specialists (unlike our heterogeneous sample), they reported similar results regarding low levels of knowledge with positive attitudes towards implementation.

It is likely that following the implementation of pharmacogenomics into clinical practice, there will be a need for continuing education and training. A study by researchers at the Mayo Clinic assessed clinicians’ reactions to pharmacogenomic clinical decision support alerts integrated into electronic health records. Authors reported that 52% of physicians did not plan to use or were unsure about using pharmacogenomic information during prescribing (Sauver et al., 2016). They also report that only 30% of physicians who received an alert changed their prescription to an alternative medication, suggesting a lack of comfort in integrating results of pharmacogenomics testing in practice. A recent meta-analysis was conducted to better comprehend the needs and preferences of both patients and healthcare professionals in facilitating their understanding of information related to pharmacogenomic testing (Veilleux et al., 2020). The results revealed the recurrence of three primary themes: (a) knowledge and understanding of genetics and pharmacogenomics; (b) experience with pharmacogenomic testing and (c) educational resources.

Finally, several barriers to the implementation of pharmacogenomics have been reported in the literature, including low familiarity among healthcare professionals, as well as technical and logistical challenges (Krebs & Milani, 2019). A previous UK qualitative study was conducted to explore barriers, challenges, and opportunities to pharmacogenomics implementation in primary care using semi-structured interviews (Rafi et al., 2020). The study included 18 participants, among which 89% were general practitioners. The majority expressed reservations about the cost-effectiveness of implementing pharmacogenomics in primary care. This is similar to our focus group findings where cost was considered a possible barrier to the implementation.

One of the strengths of our study is the large sample size which aids in the generation of representative data about the target population and the extrapolation to healthcare professionals in tertiary care settings. Our study is also the first to include nurses who are vital team members and will play a role in pharmacogenomic testing and patient education. Furthermore, this research addresses a gap in the limited literature on clinical pharmacogenomics in the Middle East. Finally, applying a mixed-methods design provides robust data, facilitates a comprehensive understanding of the research question, and enables triangulation to enhance the validity of findings. Our study, however, is not without limitations. One of the limitations is that the sample was drawn exclusively from tertiary healthcare settings, and responses from healthcare professionals in primary or secondary care settings may differ. Furthermore, we utilised convenience sampling in the quantitative phase of the study, which might have introduced self-selection and response bias, which can affect the generalisability of the findings. Future studies should apply more robust sampling such as random sampling or multi-stage systematic random sampling techniques. Another limitation was the limited number of physicians in the focus groups. Despite this, the results of the focus group discussions remained consistent with the survey results which included responses from 159 physicians. However, having more physician participants might have contributed additional insights and perspectives on the real-world application and potential barriers to pharmacogenomics implementation.

In conclusion, although healthcare providers generally recognise the benefits of pharmacogenomics, a significant knowledge gap remains. Pharmacists with a PharmD degree scored higher on knowledge assessments compared to other healthcare professionals. When evaluating readiness for implementation, most providers reported low confidence, with barriers identified as insufficient knowledge, lack of specialised training, and absence of clinical guidelines.

## Data Availability

The datasets used and/or analysed during the current study are available from the corresponding author on reasonable request.
